# Ferrocene-Based Compounds with Antimalaria/Anticancer Activity

**DOI:** 10.3390/molecules24193604

**Published:** 2019-10-07

**Authors:** Sijongesonke Peter, Blessing Atim Aderibigbe

**Affiliations:** Department of Chemistry, University of Fort Hare, Alice campus, Eastern Cape 5700, South Africa; 201414787@ufh.ac.za

**Keywords:** organometallics, malaria, cancer, ferrocene compounds, ferroquine

## Abstract

Malaria and cancer are chronic diseases. The challenge with drugs available for the treatment of these diseases is drug toxicity and resistance. Ferrocene is a potent organometallic which have been hybridized with other compounds resulting in compounds with enhanced biological activity such as antimalarial and anticancer. Drugs such as ferroquine were developed from ferrocene and chloroquine. It was tested in the 1990s as an antimalarial and is still an effective antimalarial. Many researchers have reported ferrocene compounds as potent compounds useful as anticancer and antimalarial agents when hybridized with other pharmaceutical scaffolds. This review will be focused on compounds with ferrocene moieties that exhibit either an anticancer or antimalarial activity.

## 1. Introduction

Malaria is a lethal disease and it is caused by a parasitic protozoan of genus *Plasmodium*. There are four *Plasmodium* parasites causing malaria in humans: *P. falciparum, P. vivax, P. malariae,* and *P. ovale*. Among the aforementioned parasites, *P. falciparum* is the most dangerous and it is responsible for over 95% malaria infections worldwide [1,2]. According to a WHO report, over 200 million cases and over 400,000 deaths were reported in 2016 [3]. Young children who are less than five years old and pregnant women were the major victims of malaria infections [1,2]. The most common symptoms that are associated with malaria are fever, fatigue, headaches, vomiting. In serious cases of malaria, symptoms such as yellow skin, seizures, and death have been reported [1,2,3]. 

Cancer is also a life-threatening disease involving a rare cell growth which can spread to body organs. Many cancers are caused by genetic mutation (90–95%) and some are caused by inherited genetics (5–10%) [4]. Cancer was accountable for more than 9 million deaths in 2018 especially in Africa and in Asia, according to the World Health Organization [5]. Cancer can affect any body tissue and it is named according to the body organs it affects e.g., lung, liver, breast, colorectal, and stomach cancer [6]. Among the aforementioned cancer types, lung cancer is accountable for the highest number of deaths (1.8 million) because of limited prognosis, followed by colorectal (881,000), stomach (783,000), and liver (782,000), while breast cancer (627,000) is ranked the fifth since its prognosis is favorable [5].

In the treatment of cancer and malaria, the challenges with the currently used drugs are drug toxicity, high cost, and drug resistance [7,8]. Ferrocene compounds have been described as potential compounds with unique antimalarial and anticancer activity [7,8]. In this review, the efficacy of compounds containing ferrocene moiety in vitro and in vivo are reported.

## 2. Ferrocene (Biological Activity)

Ferrocene was first discovered accidentally in the early 1950s by two researchers called Kealy and Pauson at Duquesne University while reacting C**_5_**H**_5_**BrMg with FeCl_3_ [9]. Ferrocene is useful in the modern organometallic chemistry industry due to its versatile applications in catalysis, material sciences, medicinal chemistry, and diagnostic applications [10,11]. It has been proven that the combination of organometallic compounds with known antimalarial drugs can result in potent antimalarials. Ferrocene is a good pharmacophore that displays physicochemical properties that have favorable effects on living matter [2,12].

Presently, some researchers have designed hybrid compounds containing ferrocene with several biological activities [2,12]. Antimalarial, anticancer, antitumor, antifungal, and antileishmanial activities are some biological activities exhibited by ferrocene derivatives [2,12]. Hybrid compounds in which ferrocene derivatives are linked to other compounds via selected linkers have been found to increase the efficacy of the compounds hybridized with ferrocene derivatives, resulting from its properties such as good stability to water and air, low toxicity, unfamiliar redox activity, similarities in aromaticity, and chemical versatility [13,14]. The introduction of substituent on the cyclopentadiene ring of ferrocene contributes to its biological activity [15]. The replacement of an aryl or heteroaryl core with a ferrocene core in a bioactive compound contributes to significant changes in the properties of the compounds, such as solubility, hydrophobicity, and lipophilicity [10].

Ferrocene derivatives have been reported to exhibit antiproliferative activity against several cancer cell lines with low toxic effects when compared to known anticancer agents. Its complex also exhibits distinct oxidation-reduction behavior whereby it converts readily to its one-electron oxidation product, the ferricenium ion, a radical cation of eminent stability. The aforementioned reaction is reversible with the inherent electron transfer useful for biological reactions. Its electron transfer and free radical reactions are useful in biological processes. Some of the important biological reactions of ferrocene analogues are ferricenium cation reduction by NADH and metalloproteins; enzymatically oxidation of ferrocene by hydrogen peroxide; recombination of the ferricenium system with an attack on free radicals leading to substituted ferrocene upon proton elimination [16].

Ferricenium cation react with biologically important superoxide anion radicals, resulting in the regeneration of ferrocene and dioxygen. The oxidation of the ferrocene complex to its ferricenium counterpart is feasible whereby ferrocenylcarboxylates interact with the aggressive hydroxyl radical, transforming it into harmless hydroxyl anion. In cancer etiology, free-radical chemistry plays a vital role in the different phases of growth and control of neoplasia [16]. 

The superoxide and the free radical-scavenging reaction of ferrocene are useful in the inhibition of cancer growth. Furthermore, the deactivating recombination of ferrocene in its oxidized state with the free-radical form of ribonucleotide reductase, an important enzymatic link in DNA synthesis, makes ferrocene a useful scaffold for the development of potent compounds [16]. The aforementioned factors have prompted research on the development of ferrocene derivatives with enhanced biological activity. Köpf-Maier et al. reported ferricenium salts with high biological activity influenced by their good water solubility. The water insoluble ferricenium salts did not exhibit any biological activity [17] Neuse further reported ferrocene-based anticancer drugs which were highly water soluble with excellent anticancer activity [17,18].

## 3. Ferrocene-Based Compounds with Antimalaria Activity

Each antimalarial drug is characterized by a unique mechanism of action [19]. Antimalarial drugs are classified according to the stages of the malarial life cycle in which they act, or based on their structures [20]. They are classified according to the stages of malaria life cycle such as tissue schizontocidal drugs e.g., pyrimethamine (i.e., they act on the erythrocytic stage); hyponozoitocidal drugs e.g., primaquine (i.e., those that act at the exo-erythrocytic stage); blood schizontocidal drugs e.g., quinine; and gametocytocides e.g., chloroquine [19]. They are classified based on their structures such as aryl amino alcohols e.g., halofantrine, quinidine, quinine, mefloquine; 4-aminoquinolines e.g., amodiaquine, chloroquine; folate synthesis inhibitors e.g., sulfonamides, sulfones, proguanil, chloroproguanil; diaminopyrimidine, pyrimethamine; 8-aminoquinolines e.g., primaquine; antimicrobials e.g., fluoroquinolones, doxycycline, clindamycin, tetracycline, azithromycin; peroxides e.g., artemisinin derivatives; naphthoquinones e.g., atovaquone; and iron chelating agents e.g., desferrioxamine [21]. However, the use of a single antimalarial drug for the treatment of malaria is hampered by drug resistance [22]. Malaria is treated by combination therapy in which two or more antimalarials are combined together. However, the aforementioned approach also suffers from challenges such as drug–drug interaction, drug resistance indicating the serious need to develop effective antimalarials [23]. Some researchers have reported the efficacy of hybridizing ferrocene derivatives with known antimalarials resulting in potent antimalarials.

### 3.1. Ferrocene-Quinoline Derivatives 

**Figure 1:**
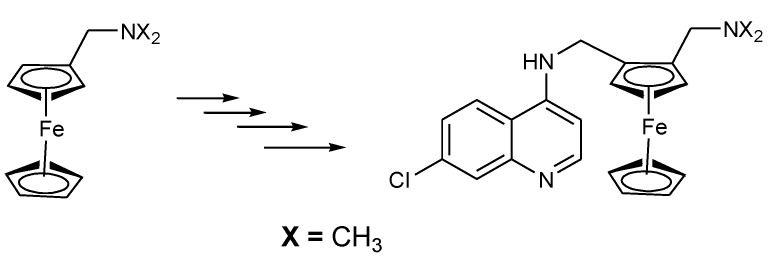
Synthesis of ferroquine by Biot and co-partners.

The strategy of hybridizing ferrocene moieties with active antimalarial agents such as chloroquine was first reported in the early 1990s and there are more studies which are ongoing. Ferroquine was derived from chloroquine in 1994 by Biot and co-workers at Lille University. It was reported to be more effective against chloroquine resistance *P. falciparum* and non-toxic in vivo [18]. Ferroquine was synthesized by the incorporation of ferrocene moiety into chloroquine whereby the methylene group of chloroquine was replaced by a ferrocene moiety (Figure 1) [18].

In vitro evaluation of ferroquine against *P. falciparum* strains revealed no correlation between ferroquine and chloroquine responses using standardized initial parasitaemina during the assays. In vivo evaluation reported by Long et al. on *Plasmodium*-infected mice revealed that a dose of ferroquine (10 mg/kg/d for four days) was suitable for effective treatment [18,24]. Ferroquine exhibits similar properties as its parent drug, chloroquine. However, the basicity and lipophilicity of ferroquine are different from that of chloroquine. The protonation of chloroquine and ferroquine at food vacuole pH resulted in lipophilicity of log D = −1.2 and −0.77, respectively which was close. However, protonation of chloroquine and ferroquine at pH 7.4 resulted in lipophilicity of log D = 0.85 and 2.95, respectively. Ferroquine exhibited lower pKa values when compared to chloroquine suggesting low vascular accumulation of ferroquine when compared to chloroquine. However, the electron donating property of ferrocene and the strong hydrogen bond between the terminal nitrogen atom and the 4-amino group may have contributed to its low pKa values. Although ferroquine has low pKa values, it exhibits a significant inhibition effect against beta-haematin formation when compared to chloroquine and it is preferentially localized at the lipid–water interface, making it an effective antimalarial agent when compared to chloroquine [18,25]. 

**Figure 2:**
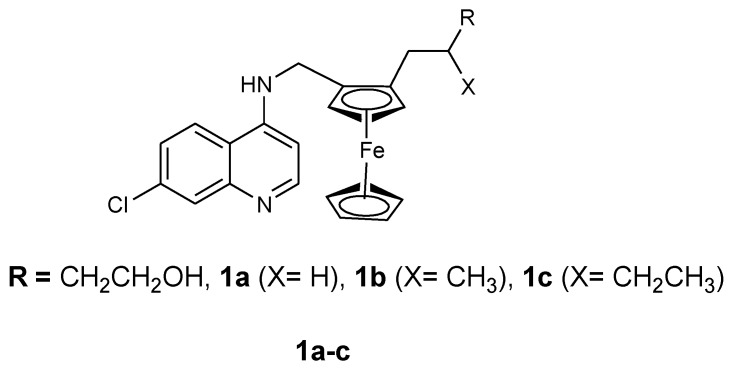
Chemical structure of compound hydroxyferroquine derivatives (**1a**–**c**).

**Figure 3:**
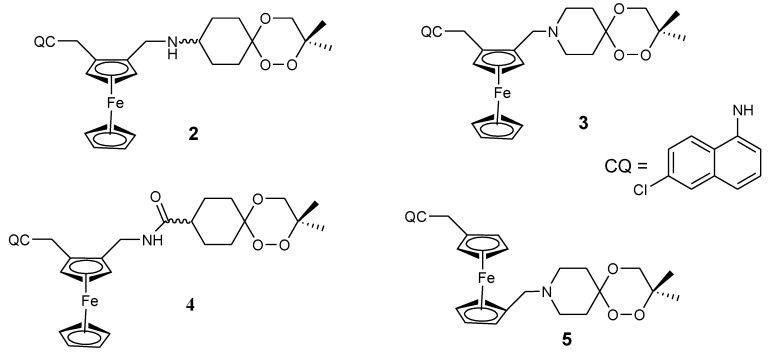
Chemical structures of trioxaferroquine derivatives (**2**–**5**).

Ferroquine enhanced antimalarial activity in vitro and in vivo against *P. falciparum* has prompted several researchers to develop antimalarial drugs containing ferrocene moiety. They have used several strategies to develop ferroquine derivatives such as hydroxyferroquine, trioxaferroquine, and chloroquine-bridged ferrocenophane with the hope of increasing the efficacy of ferroquine. Biot et al. synthesized 3-hydroxyferroquine derivatives (**1a**–**c**) which inhibited the growth of *P. falciparum* in vitro when compared to chloroquine (Figure 2). However, they were less active when compared to ferroquine. In vitro evaluation was performed against 3D7 and 2W strains of *P. falciparum* for compounds **1a**–**c** as shown in Table 1 [18,26]. Derivatives of trioxaferroquines (**2–5**) were reported by Bellot et al. (Figure 3). They were effective against chloroquine-resistant strains (FcB1 and FcM29) with IC_50_ values between 16–43 nM [18,27]. Compound **2** exhibited significant antimalarial activity with IC_50_ values 17 and 29 nM against FcB1 and FcM29, respectively. In vivo studies on *P. vinkei* pentteri infected mice at a daily oral dosage of 10 and 25 mg/kg/d over a period of 30 days were evaluated. The mice administered 10 mg/kg/d exhibited parasitemia below detectable levels with no recurrence. Mice administered 25 mg/kg/d exhibited parasitemia below detectable level between 1–18 days but displayed some recurrence between day 17 and 21. Trioxaferroquine was very effective in vivo at low dosages (10 mg/kg/d) when compared to the high dosage (25 mg/kg/d). No significant curative effect with the absence of recurrence was not achieved at 25 mg/kg/d dosage [18,27]. 

**Figure 4:**
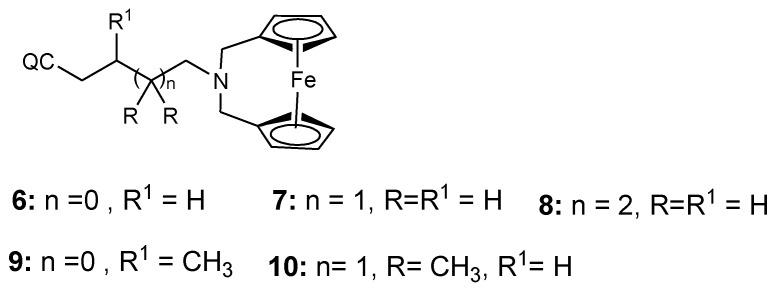
Hybrid compounds **6**–**10**.

Salas et al. synthesized ferroquine derivatives (chloroquine-bridged ferrocenophane) with two ferrocene rings bridging the terminal nitrogen atoms of chloroquine (**6**–**10**) (Figure 4). Comparison studies between the chloroquine-bridged ferrocenyl derivatives and monosubstituted ferrocenyl analogues which indicated that the monosubstituted ferrocenyl analogues capacity to retain their antimalarial activity against the drug-resistant strains was significant. Their enhanced antimalarial activity was attributed to the presence of an intramolecular hydrogen bonding. However, the structure and a balance between the lipophilicity and hydrophilicity of the bridged compounds also contributed to their unique structural capability to escape the mechanisms of resistance [18,28].

**Figure 5:**
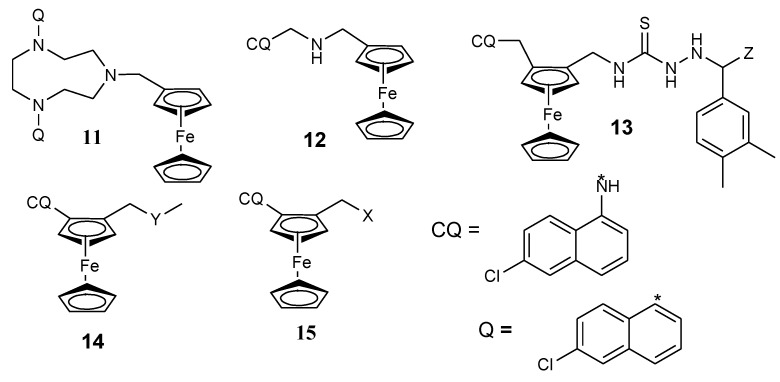
Chemical structures of quinolone-ferrocene hybrids (**11**–**15**). **13**-Z = CH_3_, **14-Y** = 1,2,3,5-diisopropylidene glucofuranose moiety, **15a**-X = diisopropylidene-protected 6-amino-deoxyglucofuranose, **15b**-X = 6-amino-6-deoxygalactopyranose.

Biot et al. also reported a ferrocene-triazacyclononane quinolone conjugate (**11**) (Figure 5) with potent antiplasmodial activity against chloroquine strain Dd2 of *P. falciparum* in vitro [20,29]. Dormale et al. synthesized a ferrocene derivative from ferrocene and 4-aminoquinolines. In vitro, the compound prepared by modification with tartaric acid was the most potent compound. Its antimalarial activity was significant at low concentrations when compared to chloroquine against chloroquine-susceptible strain SGE2 and the chloroquine-resistant strains FCM6 and FCM17. The compound was able to inhibit the resistance of parasites. The compound’s capability to inhibit the efflux mechanism which is common with chloroquine may have contributed to its enhanced antimalarial activity when compared to chloroquine [19,20,30]. A class of quinoline–ferrocene hybrids were reported by N’Da et al. Hybrids with flexible linkers were effective when compared to compounds with rigid linkers which were ineffective against D10 and Dd2 strains of *P. falciparum.* The compound with the 3-aminopropyl methylamine linker (**12**) exhibited good antiplasmodial activity (19-fold) in vitro when compared to chloroquine with IC_50_ values of 0.008 versus 0.148 μM against the Dd2 strain of *P. falciparum* [20,31].

Biot et al. reported chimeras of ferroquine and thiosemicarbazones (**13**). In vitro studies on strains of *P. falciparum* and parasitic cysteine proteas, falciparum-2 revealed that the antimalarial activity of the compounds was significant because of the presence of aminoquinoline which made the compound easily conveyed to the digestive vacuole of the parasite [20,32]. David et al. synthesized quinolone ferrocene ester and evaluated it against chloroquine strains of *P. falciparum* (Dd2 and D10). The esters prepared from ferrocenylformic acid exhibited antimalarial activity against Dd2 and D10 strains of the *P. falciparum*. However, their antiplasmodial activity against D10 strain was not high when compared to the chloroquine-sensitive Dd2 strain. The ester with a butyl branch linked to the ferrocene moiety was very effective against all the strains with an IC_50_ of 0.13 µM on the resistant strain and 2.5-fold higher activity when compared to chloroquine with an IC_50_ of 0.34 µM. The compounds were highly selective towards *P. falciparum* [15,33]. 

Herrmann et al. prepared two derivatives of ferrocene hybrids containing 1,2,3,5-(diisopropylidene)-α-d-glycofuranose moiety combined with chloroquine derivative (**14**) with good antimalarial activity when tested against two strains of *P. falciparum* (Dd2 and K1) (Figure 5) [20,34]. The second class of Herrmann et al. hybrids was prepared by conjugation of ferrocene scaffolds with 7-chloroquinoline via an ether linker followed by the attachment of diisopropylidene protected 6-amino-6-deoxyglucofuranose or 6-amino-deoxygalactopyronose using reductive amination resulting in the isolation of compound **15a** and **15b**. Both compounds exhibited good antimalarial activity with an IC_50_ value of 0.77 µM resulting from the presence of the carbohydrate moiety. The resistance indices for the compounds were less than 1, indicating a high activity against the Dd2 strain when compared to the D10 strain [20,35].

Chavain et al. conjugated ferroquine analogues with glutathione reductase inhibitor via a cleavable amide bond for targeting different pathways of the malaria parasite. The analogues antimalarial activity in vitro against NF54 (sensitive) and K1 (resistant) strains of *P. falciparum* was not significant, suggesting that their mode of action differs from ferroquine and chloroquine modes of action. Their poor antimalarial activity is also attributed to the cleavage of the amide bond and the side chain when metabolized in the digestive vacuole of the parasite. The aforementioned findings reveal how the design of molecules can influence their antimalarial activity [20,36].

### 3.2. Artemisinin-Ferrocene Derivative

**Figure 6:**
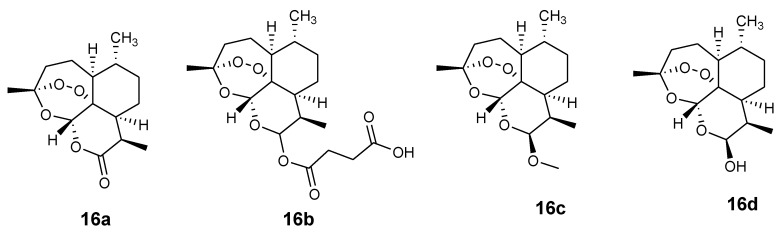
Artemisinin and its derivatives.

Artemisinin-based derivatives **16a**–**d** (artemisinin, artesunate, artemether, and dihydrortemisinin, respectively) are characterized by fast action and a short half-life (Figure 6). Artemisinin-based combination therapies (ACT) are promising therapeutics effective in controlling *P. falciparum* drug resistance. Synthesizing hybrid drugs containing artemisinin derivatives and other effective drugs such as ferroquine and chloroquine is a good approach to develop potent antimalarials that can overcome drug resistance. Ferroquine combined with artesunate has been reported to be a promising antimalarial therapeutic which was safe at all doses tested in vivo [37,38]. 

**Figure 7:**
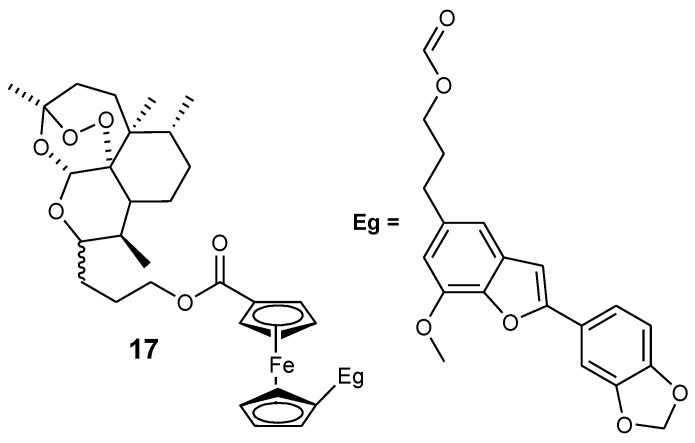
Hybrid compound **17**.

**Figure 8:**
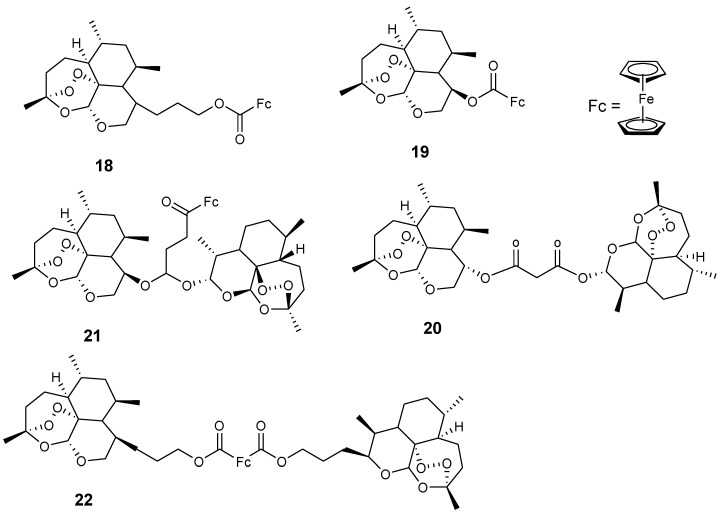
Chemical structures of second generation dihydroartemisinin ferrocene derivatives (**18**–**22**).

Reiter et al. reported an artemisinin derivative containing ferrocene moiety and egonol (**17**). In vitro studies indicated that the derivative **17** was the only one exhibiting antimalarial activity among the seven artemisinin–ferrocenyl derivatives studied while others exhibited anticancer activity. Its antimalarial activity was enhanced when compared to the parent drug, egonol, with an inhibition value of 88 nM (Figure 7) [37,39]. Reiter et al. also reported a second generation of 1,2,4-trioxane-ferrocene derivatives (**18**–**22**), and evaluated their antiplasmodial activity against the 3D7 strain of *P. falciparum* using chloroquine and dihydroartemisinin as controls (Figure 8). These trioxane–ferrocene compound antiplasmodial activities were significant with IC_50_ values in the range of 7.2–30.2 nM. Compound **20** IC_50_ value was 7.2 nM and promising when compared to chloroquine. Compound **18** and **19** IC_50_ values were 8.6 and 13.4 nM, respectively [37].

**Figure 9:**
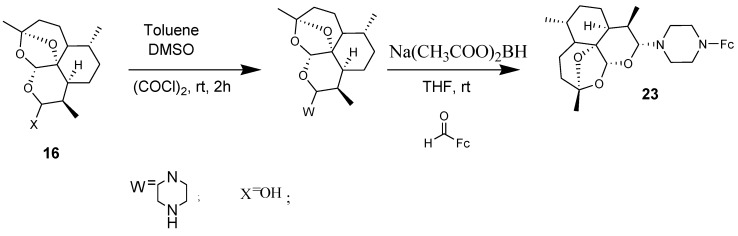
Synthesis of amino–artemisinin–ferrocene compound (**23**).

Amino-artemisinin-ferrocene **23** in which ferrocene was incorporated via a piperazine linker to dihydroarteminisinin derivatives was tested in vitro against chloroquine-sensitive NF54 and chloroquine-resistant K1 and W2 strains of *Plasmodium* falciparum parasites (Figure 9). The compound exhibited good activity against the asexual parasites with IC_50_ values of 2.79 and 3.2 nM against K1 and W2 strains of *Plasmodium* falciparum parasites, respectively. The resistance indices further indicated the compound reduced capability for cross resistance [40]. 

**Figure 10:**
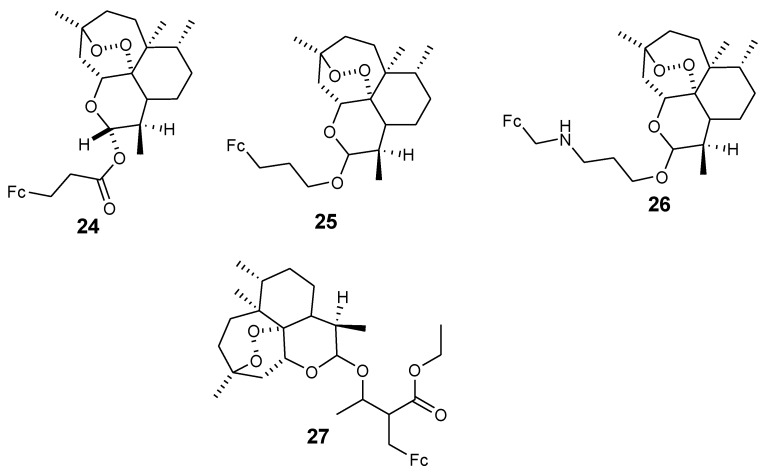
Chemical structures of dihydroartemisinin ferrocene derivatives (**24**–**27**).

Delhaes et al. synthesized ferrocene–artemisinin derivatives (**24**–**27**) from ferrocene derivatives and dihydroartemisinin in good yield in the range of 60–80% (Figure 10). In vitro studies using HB3 and SGE2 strains (sensitive) and Dd2 strain (resistant) of *P. falciparum* showed that compound **26** was the most effective antimalarial. In vitro evaluation showed that its IC_50_ were similar to those of artemisinin (HB3 = 12 nM, SGE2 = 11 nM, and 14 nM against Dd2) [41]. The compound’s capacity to bind with ferroprotoporphyrin IX was significant. These effect of adducts between artemisinin derivatives on heme in the generation of artemisinin radicals was significant. 

### 3.3. Ferrocene–Novobiocin Derivatives

Novobiocin is an antibiotic that is produced by bacteria called *Streptomyces,* its inhibitor of the chaperone, heat shock protein 90 (Hsp90) which is responsible for the stabilization of proteins and it is found to be one of the antibiotics that are biological active with anticancer and antimalarial activity when it is incorporated together with other active molecules such as ferrocene [42,43,44]. 

**Figure 11:**
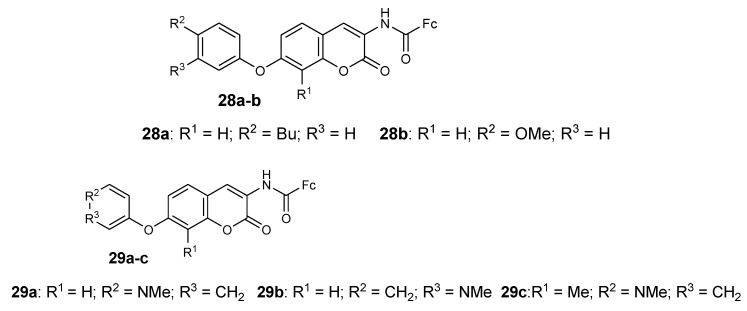
Ferrocenyl–novobiocin derivatives (**28a**–**b** and **29a**–**c**).

Novobiocin derivatives containing ferrocene moieties were synthesized and documented by Mbaba et al. and they were tested against the 3D7 strain (sensitive) of *P. falciparum* in vitro (Figure 11). Compounds **28a** and **28b** exhibited moderate antimalarial activity when compared to other synthesized compounds which exhibited poor antimalarial activity when tested against the 3D7 *P. falciparum* strain with an inhibition value of 9 nM in vitro [43]. The incorporation of ferrocenyl moiety on the benzamide side of novobiocin resulted in compounds with enhanced biological activity. Furthermore, the cell viability studies indicated that the inhibition growth of *P. falciparum* by the compound did not exhibit toxic effects, which was significant with HeLa cell viability which was greater than 70%. It also indicated that the compounds were highly selective towards the parasitic cells when compared to the human cells [43].

Mbaba et al. reported another series of ferrocene–novobiocin derivatives and evaluated them for antiplasmodial activity and human cytotoxicity using the 3D7 strain of *P. falciparum* and HeLa cells, respectively, in vitro. Compound **29a**–**c** which contain the *N*-methyl group in the piperidine ring exhibited better antimalarial activity with IC_50_ values that were below 10 μM but compound **29c** was the most potent compound with an IC_50_ value of 0.889 μM [44]. It was reported that these three compounds were selective towards the *P. falciparum* parasite and their HeLa cell viability was below 25% with no toxic effects [44]. Novobiocin and other derivatives from this series of compounds were inactive with HeLa cell viability that was greater than 75%, which means N-methyl substituents play an important role in the antiplasmodial activity of these compounds and also these compounds were found to be independent of Hsp90 [44].

### 3.4. Ferrocene- Pyrrole Derivatives

**Figure 12:**
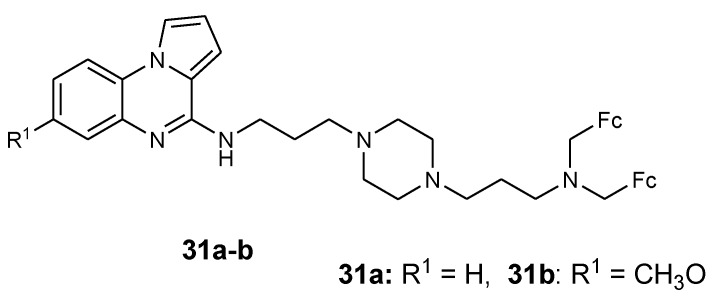
Structure of synthesized ferrocenyl pyrrolo[1,2-a]quinoxaline derivatives (**30a**–**b** and **31a**–**b**).

Guillon et al. reported the synthesis of ferrocenic pyrrolo[1-2-a]quinoxaline derivatives from nitroanilines using different strategies (**30a**–**b** and **31a**–**b**) (Figure 12). Compounds **30a**–**b** were synthesized via regio-selective palladium catalyzed monoamination and compound **31a**–**b** was prepared by reductive amination strategy. These derivatives (**30a**–**b** and **31a**–**b**) were tested against FcB1 and PFB (resistant) and F32 (sensitive) strains of *P. falciparum* in vitro [45]. The antimalarial activity of the compounds was dependent on the nature of the substituent on the bis(3-aminopropyl)piperazine linker. The pyrrolo[1,2-a]quinoxalines with nitro substitutions were the most active compounds against the CQ-sensible F32 strain (IC_50_ 0.038–0.085 mM). The presence of a 4-methoxy substituent on the benzyl terminal group also enhanced the antimalarial activity against F32 strain with IC_50_ 0.045 mM. The compound with a 5-nitro-2-hydroxybenzyl substituent on the bis(3-aminopropyl)piperazine chain exhibited the most potent antimalarial activity with IC_50_ of 0.048 and 0.060 mM, respectively, against CQ-resistant FcB1 and K1 strains. Compounds with NO_2_ substituents inhibited β-hematin formation significantly when compared to chloroquine [45].

### 3.5. Isatin–Ferrocene Conjugates

**Figure 13:**
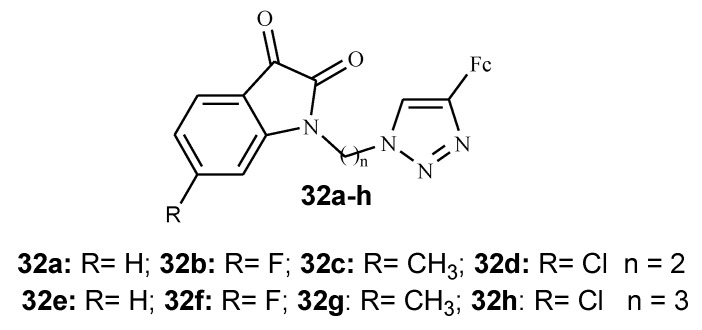
Chemical structure of isatin–ferrocene-based derivative (**32a**–**h**).

Isatin is a heterocyclic scaffold that is isolated from the leaves and roots of *Couroupita guianesis* and *Calanthe discolour, Isatis tinctoria* and it is found to be versatile in organic synthesis because of the many possibilities of modification at C-3, C-5, and N-1 with various activities such as anticancer, antimalarial, and antifungal [46,47,48]. Kumar et al. synthesized several isatin–ferrocene conjugates tethered with 1*H*-1,2,3-triazole derivatives (**32a**–**h**) via copper-promoted azide–alkyne cycloaddition reaction and tested them against chloroquine-resistant strains (3D7 and W2) of *P. falciparum* (Figure 13) [47]. The presence of a halogen substituent on the C5 position of the isatin ring and a propyl linker influence the antimalarial activity of the compounds [47].

Compound **32a**–**d** with *n* = 2 (ethyl) as a linker did not exhibit antimalarial activity even at higher concentrations. With compound **32e**–**h** with *n* = 3 (propyl) as a linker, antimalarial activity showed improvement with IC_50_ values ranging between 3.76 and 16.20 μM indicating that the chain length has an effect on the activity of these compounds. When compared with ferroquine (2.1–13.4 nM), in vitro antimalarial activity of compound **32e**–**h** was lower. From the series of isatin–ferrocene derivatives, only compounds (**32f**–**h**) with the electron withdrawing groups and propyl as a chain linker displayed good antimalarial activity with IC_50_ values of 3.76 and 4.58 μM against chloroquine-resistant strains (3D7 and W2) of *P. falciparum*. Further evaluation of compounds (32**f**–**h**) for cytotoxicity against HeLa mammalian cells revealed their non-cytotoxic effect and their selective inhibition of *P. falciparum* [47].

### 3.6. Ferrocene–Pyrimidine Conjugates

**Figure 14:**
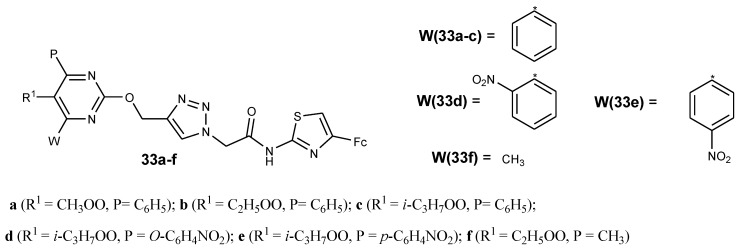
Schematic route showing the synthesis of ferrocene-pyrimidine conjugates (**33a**–**f**).

Recently, researchers reported that pyrimidine moiety linked with ferrocene resulted in hybrid compounds with good antiplasmodial activity against chloroquine strains of *P. falciparum*. In vitro antiplasmodial analysis showed that compounds **33a**–**f** were active against the chloroquine-susceptible NF54 strain (Figure 14). The pyrimidine ring substituition of methyl ester group at C-5 of compound **33a** with an ethyl or Iso-propyl group to yield compound **33b** and **33c** increased the lipophilicity of the compounds. Increasing the lipophilicity enhanced the antiplasmodial activity of the drug. For compound **33c**–**e**, the antiplasmodial activity decreased in the order **33c** > **33d** > **33e** due to the decrease in lipophilicity. Compounds substituted with different functional groups (**R_1_** and **P**) at position C-4 and C-5 of pyrimidine exhibited good antimalarial activity with the lipophilicity playing a major role, and the reversible oxidation behavior of these hybrid compounds was similar to that of ferrocene [49].

### 3.7. Ferrocenyl Chalcones

**Figure 15:**
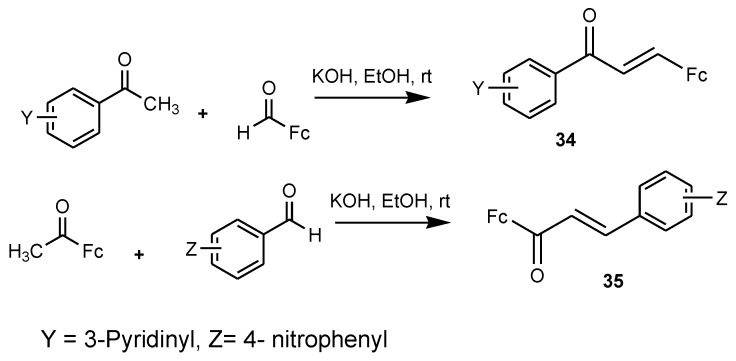
Ferrocenyl chalcones derivatives (**34**–**35**).

Chalcones are prepared from an aromatic ketone group and they exhibit several biological activities such as antimalarial and antitumor activity. Licochalcone from the licorice root in China has been reported to be a promising antimalarial agent. Synthesis of ferrocenyl chalcones was obtained by replacing the aromatic ring with a ferrocenyl moiety [50,51,52,53]. Researchers synthesized several ferrocenyl chalcone derivatives by base-catalyzed Claisen–Schmidt condensation. However, only two compounds (**34** and **35**) were reported to be effective antimalarial agents, with low IC_50_ values of 4.5 µM (A) and 5.1 µM (B) against chloroquine-resistant strains of *P. falciparum* in vitro (Figure 15) [52]. The position of the substitution of nitro and pyridine rings influenced the biological activity of the compounds. Compound **35** exhibited higher selective indices when compared to compound **34** when tested on KB and MDCK cells of *P. falciparum* (SI = 37 and SI = 14), respectively. For compound **34,** the selective indices were between 5 and 9 [52].

**Figure 16:**
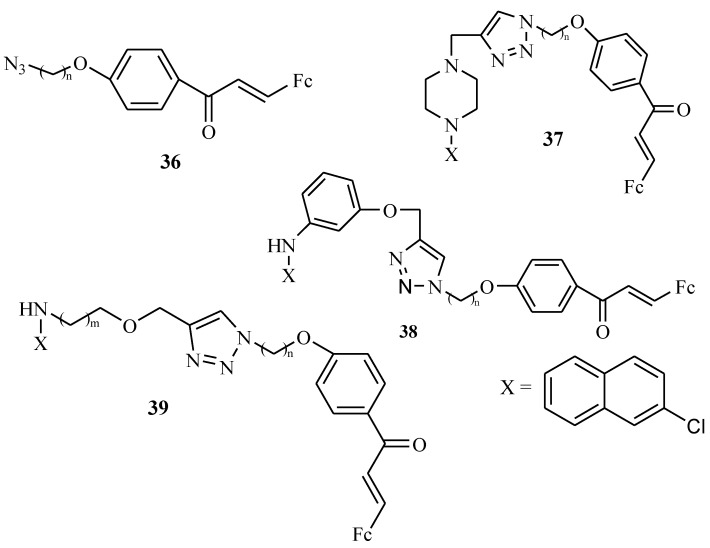
Chemical structures of *O*-alkyl azide ferrocenylchalcones (**36**); piperazine (**37**), phenyl (**38**), amino alcohol (**39**) –linked 7-chloroquinoline-ferrocenylchalcone derivatives.

Kumar et al. reported the synthesis of 1*H*-1,2,3-triazole tethered 4-aminoquinoline-ferrocenylchalcone derivatives (compounds **37**, **38**, and **39** with compound **36** acting as a linker) (Figure 16) and tested them for antimalarial activity against *P. falciparum* chloroquine-resistant (W2) strains in vitro. Different strategies were used in the synthesis of compounds **37**, **38**, and **39**. They were prepared by Cu-promoted azide–alkyne cycloaddition for both compound **37** and **38**. Compound **39** was prepared by Huisgen’s azide–alkyne cycloaddition. These derivatives **37**–**39** were tested in vitro for antimalarial activity against the W2 (resistant) strain of *P. falciparum* and they exhibited good antiplasmodial activity with inhibition values ranging between 0.37–5.08 µM. The alkyl chain length influenced the antimalarial activity of the compounds [52]. In compounds **37a**–**f** with a piperazine ring, the length of the chain had no effect on compounds’ antimalarial activity. The IC_50_ values of compounds **37a**–**f** ranged between 2.55–5.08 µM. In compound **38**, the piperazine ring was replaced by 4-aminophenol and the length of the chain played no vital role in the biological activity of the compounds. However, (**38e**–**38f**) exhibited improved antimalarial activity when compared to compounds **37a**–**f** with inhibition values of **38e** (*n* = 6, IC_50_ = 2.40 µM) and **38f** (*n* = 8, IC_50_ = 1.16 µM). In compound **39**, the chain length displayed different trends for derivatives **39a**–**i** and the introduction of amino alcohols (amino-ethanol and amino-propanol) substituents showed improvement on the activity of compounds. Increasing the alkyl chain length (C-2 to C-5) in compounds **39a**–**f** with amino-ethanol as a substituent decreased the activity of the compound. However, increasing the alkyl chain length (C-6 to C-7) improved their activity in which compound **39a** (*n* = 2, IC_50_ = 0.95 µM), compound **39d** (*n* = 5, IC_50_ = 2.92 µM), compound **39e** (*n* = 6, IC_50_ = 0.66 µM) and **39f** (*n* = 8, IC_50_ = 0.69 µM), respectively. Compounds **39g**–**j** with amino-propanol substituents exhibited good antiplasmodial activity and the chain length linker did not increase nor decrease their antimalaria activity. All of these derivatives **37**–**39** exhibited antimalarial activity but **39g**–**j** were the most active compounds with IC_50_ values ranging between 0.37–1.78 µM against W2 (resistant) strains of *P. falciparum,* in vitro [52].

## 4. Ferrocene-Based Compounds with Anticancer Activity

Cancer is a disease characterized by uncontrolled cell division [54,55]. It is a chronic and complicated disease and it is the cause of the high death rate globally [54,55]. The most fatal and common cancers are lung, breast, colorectal, stomach, and liver cancer which are responsible for almost 92% of death cases [5]. More than 200 anticancer drugs are available but less than 5% has reached the market and the drawbacks are caused by lack of clinical trials on these synthesized drugs. Some anticancer drugs enter phase I and phase II but fail to advance through phase III of a clinical trial [55]. Drug resistance is common with most anticancer drugs and this has contributed to a high death rate among cancer patients. Anticancer drugs are grouped according to their mode of actions [56,57,58].

### 4.1. Ferrocene–Indole Hybrids

**Figure 17:**
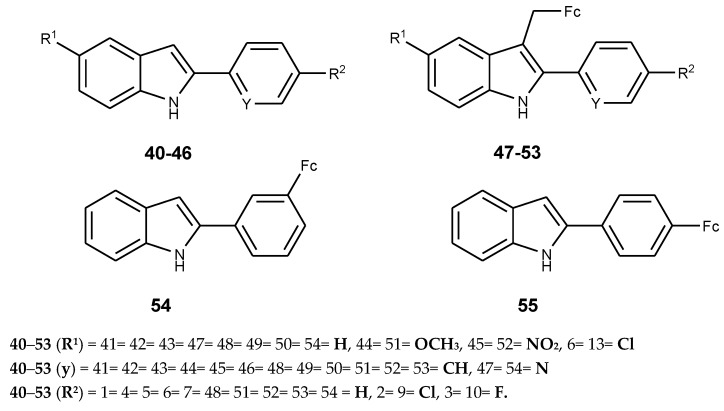
Chemical structure of indole (**40**–**46**) and ferrocene–indole derivatives (**47**–**55**).

Indoles are cheap and have unique properties with anticancer activity. Ferrocene, on the other hand, has unique chemical and pharmacological properties. Combining ferrocene and indoles derivatives into hybrid compounds is a potential route suitable for the development of effective anticancer drugs [55,56,57]. Quirante et al. synthesized ferrocenyl–indole derivatives with ferrocene moiety attached on C-3 of the 2-phenylindole skeleton (**40**–**46**) and tested for anticancer activity using a A549 human lung carcinoma cell line [59]. The synthesized ferrocenyl–indole derivatives are (**47**–**53**) (Figure 17). Compounds **50**–**52** exhibited potent cytotoxic effect against the cancer cell lines with IC_50_ values of 5, 7, and 10 µM, respectively. Compound **50** displayed potent cytotoxic activity with an IC_50_ value of 5 µM. All the ferrocenyl–indole derivatives (**47**–**53**) were more active when compared to their parent drugs (**40**–**46**). The nature of the substituents (R^1^) on C-5 of the indole ring and halogen substituents on the para-position (R^2^) of aryl ring played a significant role on the cytotoxic effect of the hybrid molecules [59].

The order of potency for R^1^ on position 5 was: Un-substituted compounds (R^1^ = H) > 5-OMe (**50**) > 5-NO_2_ (**51**) > 5-Cl (**52**) and for halogen substituents for R_2_, the potency was: p-H (**47**) > p-Cl (**48**) > p-F (**49**), respectively. Compound **47** was two-fold more active than compound **49**, compounds **48**, **49**, and **51** were 2-fold more active than their comparable derivatives (**41**, **48**, and **44**), derivative **47** and **53** were 3-fold and 4-fold more active than comparable derivatives **40** and **46**, respectively. The most active ferrocenyl–indole compound was compound **51** and it was 25-fold more active when compared to the indole compound **43 [59]**.

Radulovic’ et al. prepared 2-(3-ferrocenylphenyl)-1*H*-indole (**54**) and 2-(4-ferrocenylphenyl)-1*H*-indole (**55**) by substituting ferrocenyl moiety into a indole derivative at position 3 and 4, respectively. In vivo studies of the compounds (**54** and **55**) on rat peritoneal macrophages for cell viability evaluation revealed that the macrophage viability was reduced by more than 50% using low concentrations of these compounds. The cytotoxic effect of the compounds was dose-dependent and a high concentration of the compounds reduced the viability of peritoneal macrophages [60].

### 4.2. 1,2,4-Trioxane–Ferrocene Hybrids

1,2,4-trioxane ferrocene derivatives were reported by Reiter et al. as potential drugs for various biological activities such as antimalarial, anticancer. A 1,2,4-trioxane-ferrocene hybrid, **23**, was reported to be the most active compound from this class against leukemia cells with IC_50_ values of 0.25 µM against CCRF-CEM cells and 0.57 µM against CEM/ADR5000 cell lines, respectively. Since compound **23** exhibited good anticancer activity, the second generation of these compounds from this class (1,2,4-trioxane-ferrocene) were synthesized (**24**–**27**). In vitro studies against leukemia cells revealed significant cytotoxic effect with an IC_50_ value of 0.13 µM when compared to dihydroartemisinin with an IC_50_ value of 0.48 µM [39,61]. The compounds with two trioxane moieties such as compound **23**, **24**, **25**, and **27** exhibited cytotoxic effects with IC_50_ of 0.25, 0.13, 0.07, and 0.08 µM, respectively, against CCRF-CEM cells. The most potent compound against CCRF-CEM cells was compound **26** with an IC_50_ value of 0.01 µM. The hybrids, **24**–**27**, were more active when compared to their parent drugs (artemisinin and dihydroartemisinin) with 3- to 50-fold more cytotoxic effects towards multidrug resistant cell lines. The molecular weight and number of 1.2,4-trioxane moieties present in these compounds played an important role in their cytotoxic effects against CEM/ADR5000 cell in vitro. Compound **25** and **27** with two moieties and a molecular weight of 800 g/mol exhibited enhanced cytotoxic effects when compared to compound **23** and **24** with one moiety and a molecular weight of 500 g/mol [39,61].

### 4.3. Ferrociphenols

**Figure 18:**
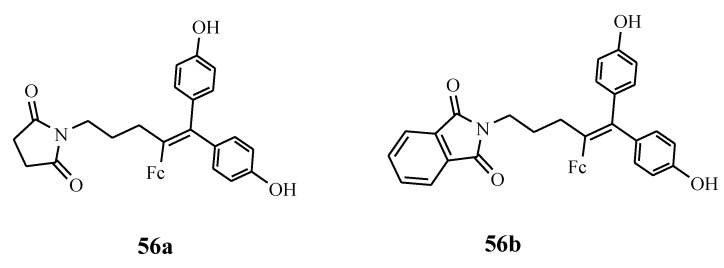
Hybrids **56a** and **56b**.

Ferrociphenols are reported as the most active antiproliferative agents when compared to cisplatin and tamoxifen cancer drugs [56]. They have rich diverse modes of action which are caused by their capability to produce a redox pattern (ferrocenyl-ene-phenol) in the cancer cell [56]. Their target is the mitochondrial system or redox proteins in the cancer cells [62,63,64,65]. Pigeon et al. prepared ferrociphenol derivatives **56a** and **56b** (Figure 18). Increasing the length of the non-polar carbon chain on the north-west side of the molecule affected the steric hindrance and lipophilicity, thereby decreasing the anticancer activity of the compounds significantly. The presence of succinimido or phthalimido substituents in some of the derivatives enhanced their antitumoral activity against ovarian cancer cell lines with low IC_50_ values which were below 0.08 µM [64].

**Figure 19:**
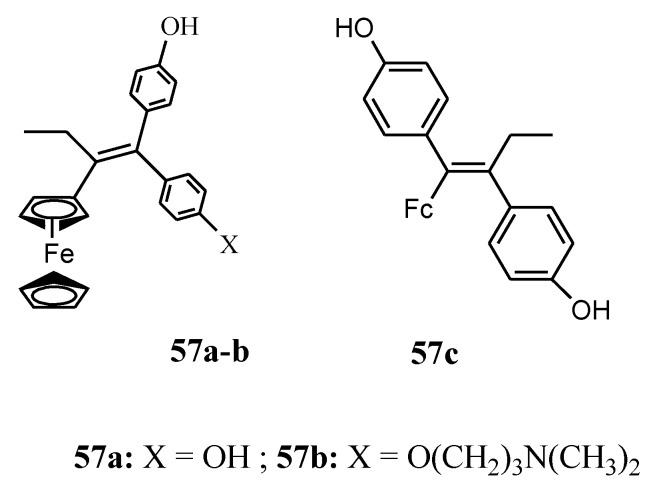
Chemical structures of ferrociphenol compound (**57a**–**c**).

Zanellato et al. evaluated the anticancer activity of compounds **57a** and **57b** against malignant pleural mesothelioma (MPM) cell lines (Figure 19). Both compounds were effective in the inhibition of cell proliferation [66]. Vassieries et al. reported the synthesis of compound **57a** and **57c [67]**. The antiproliferative effects of both compounds were evaluated at a concentration of 1 µM on hormone-dependent (MCF7) and hormone-independent (MDA-MB231) breast cancer cell lines. Compound **57c**’s antiproliferative effect was not significant when compared to **57a** which exhibited significant antiproliferative activity with IC_50_ = 0.7 µM. The result suggests that the cytotoxic activity of the ferrocenyl group is higher than the estrogenic proliferative effect of the diphenol moiety. The poor proliferative effect of **57c** on MCF7 (ER positive) cells indicate that the presence of a ferrocene group is not sufficient for the generation of antiproliferative effects and the position of the oxidizable ferrocenyl group plays a vital role in the antiproliferative activity of the compounds [67].

**Figure 20:**
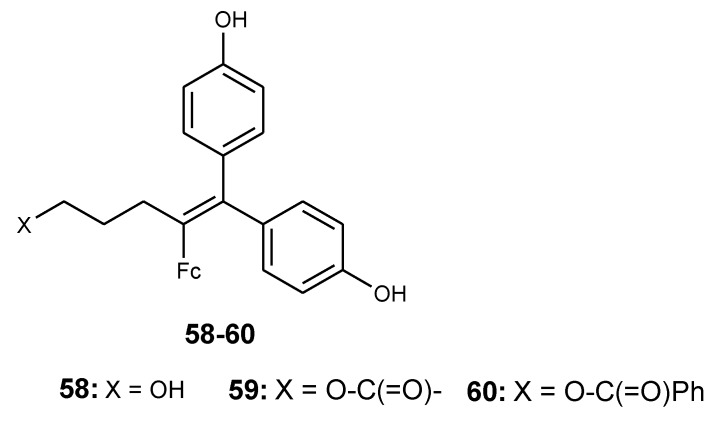
Chemical structures of hydroxypropyl-ferrocephenol derivatives (**58**–**60**).

Wang et al. reported a series of ferrociphenol derivatives with hydroxypropyl (**58**–**60**) and tested them on the triple negative breast cancer line MDA-MB-231 with IC_50_ values ranging between 0.26–13.31 Μm (Figure 20). The introduction of a bulky aromatic group at the terminal hydroxyl position resulted in compounds with poor cytotoxic activity. The replacement of terminal hydroxyl with a chlorine or propan-2-one oxime resulted in compounds that exhibited moderate cytotoxic activity against the MDA-MB-231 cells. The introduction of benzylated substituents at the terminal hydroxyl position of compound **60** resulted in significant cytotoxic effects against the MDA-MB-231 cells, resulting in a high lipophilic nature of the compound. Compound **59** and **60** exhibited significant cytotoxic effect against the cancer cell lines suggesting that the presence of an ester linker makes the compounds prone to hydrolysis, resulting in the generation of the parent drug, **58 [63]**.

**Figure 21:**
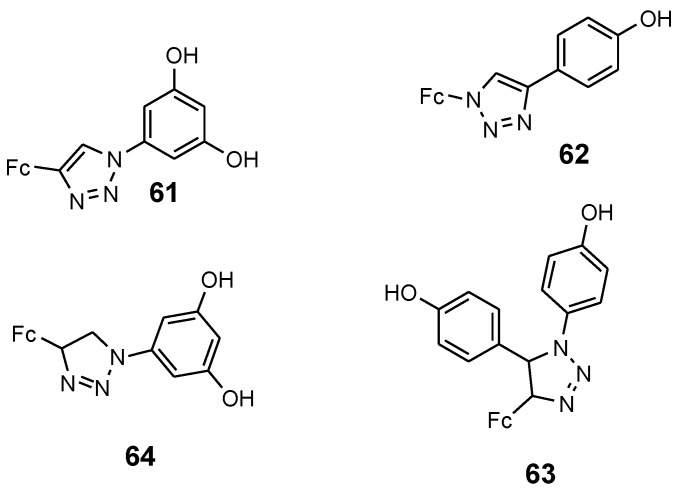
Chemical structures of hydroxyphenyl–ferrocephenol derivatives (**61**–**64**).

Plazuk et al. reported the synthesis of ferrocenyl compounds **61**–**64** with ferrociphenols and a 1*H*-1,2,3-triazolyl moiety acting as a linker and their cytotoxic activities against human breast cancer cells (HCC38 and MCF-7) in vitro (Figure 21). Some of the compounds exhibited good cytotoxic activity against the hormone-independent HCC38 breast cancer cell line. The most active compound was **61** with IC_50_ = 15.3 μM and it contained a para-hydroxyphenyl group. The introduction of two para-hydroxyphenyl moiety in compound **63** resulted in a decrease in the anticancer activity of the compound with an IC_50_ value of 30.6 μM. The introduction of a 3,5-dihydroxyphenyl in compound **64** led to a significant decrease in the anticancer activity. Compound **62** exhibited cytotoxic activity against the hormone-dependent MCF-7 cancer cell lines at high concentrations of IC_50_ = 48.9 [68].

**Figure 22:**
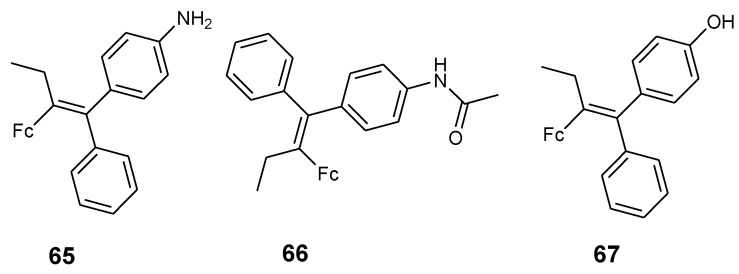
Chemical structures of 2-ferrocenyl-1-1-diphenyl-but-1-ene derivatives (**65**–**67**).

Pigeon et al. synthesized ferrociphenol derivatives (**65**–**67**) with enhanced cytotoxic activity by replacing the phenol group with an aniline or acetanilide group and these derivatives were tested against MCF-7 and MDA-MB-231 breast cancer cell lines (Figure 22). The effects of compound **65**–**67** against hormone-dependent breast cancer cells MDA-MB-231 was significant at IC_50_ values 0.8, 0.65, and 1.13 μM, respectively. Compound **65**’s proliferative effect was significant when compared to compound **66** and **67**. The effect of 65 and 66 on MCF-7 cells revealed a low cytotoxic effect at a low concentration of 0.1 μM. However, 65 exhibited enhanced proliferative effects when compared to compound 66. At a high concentration of 10 μM, compound **65** exhibited no significant cytotoxic effect but compound **66** exhibited a moderate antiproliferative effect [69].

### 4.4. Ferrocenyl Derivatives of Clotrimazole Drug

**Figure 23:**
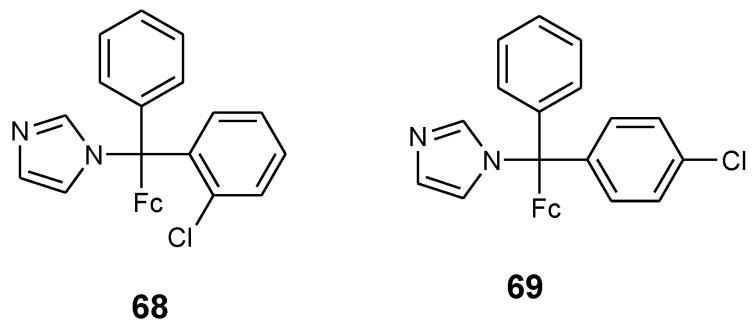
Chemical structures of ferrocenyl- clotrimazole derivatives (**68**–**69**).

Pedotti et al. reported the synthesis of two ferrocenyl–clotrimazole derivatives **68** and **69** by replacing one of the phenyl rings in the clotrimazole structure with a ferrocene moiety and studied their biological activity against two human cancer cell lines (HT29 and MCF-7) (Figure 23). In compound **68**, the chlorine was on the ortho-position whereas for compound **69** it was on the para-position. Compound **68** and **69** growth inhibition effects were studied on colorectal cancer cells (HT29) and breast cancer cells (MCF-7) and ferrocenyl–clotrimazole derivatives were more active against breast cancer cell MCF-7 when compared to the colorectal cancer cell line HT29 with GI_50_ values of HT29 = 27.51 µM, MCF-7 = 23.84 µM (**68**) and HT29 = 28.13 µM, MCF-7 = 20.44 µM (**69**), respectively. These two compounds **(68** and **69**) were more active than their parent drug (clotrimazole, GI_50_ HT29 = 64.19 µM and MCF-7 = 21.44 µM) but less active when compared to 5-fluorouracil (GI_50_, HT29 = 5.71 µM and MCF-7 = 2.98 µM). The modification of the structure with ferrocene enhanced the cytotoxic activity of the compounds when compared to the parent drug in HT29 cells. The aforementioned enhanced effect is attributed to the redox properties of iron in the ferrocene moiety and also its ability to generate cytotoxic reactive oxygen species [14].

### 4.5. Ferrocenyl Chalcogeno Triazole Conjugates

**Figure 24:**
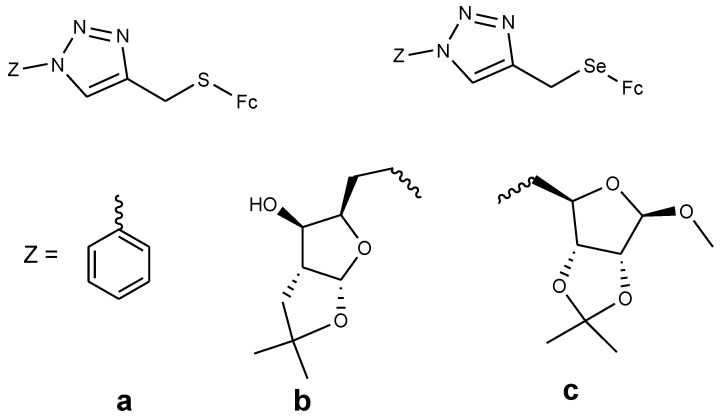
Chemical structures of ferrocenyl–chalcogeno–triazole conjugates (**70**–**71**).

Panaka et al. documented the synthesis of ferrocenyl–chalcogeno derivatives (**70** and **71a**–**c**) and tested their cytotoxic activity against five different cancer cell lines (A549, MDA-MB-231, MCF-7, HeLa, and HEK-239T) with IC_50_ values ranging between 2.9 and 20 µM for the first four cancer cells, and none of the compounds being effective against HEK-239T (Figure 24). Compound **70a**–**c** contained sulfur and compound **71a**–**c** contained selenium. The derivatives with selenium exhibited higher cytotoxicity with IC_50_ values ranging between 2.9 and 18.3 µM compared to the sulfur derivatives with inhibition values between 4.46–18.9 µM, respectively [70]. Compound **70a** exhibited good cytotoxic activity against two cancer cells (A549 and HeLa) with IC_50_ values of 11.6 µM (A549) and 14.8 µM (HeLa), **70c** and **70b** exhibited good cytotoxic activity against MDA-MB-231 cancer cells only with inhibition values of 9.7 and 18.9 µM, respectively. Compound **71a** displayed cytotoxic activity against three cancer cells with IC_50_ values of 4.56 µM (MDA-MB-231), 4.46 µM (MCF-7), and 10.9 µM (HeLa); compound **71b** which was the most cytotoxic active compound against four cancer cells with IC_50_ values of 2.9 µM (A549), 3.35 µM (MDA-MB-231), 5.58 µM (MCF-7), and 11.6 µM (HeLa). Compound **71c** exhibited high cytotoxic activity on two cancer cells with IC_50_ values of 3.71 µM (A549) and 18.3 µM (HeLa), respectively [70]. The selenium compounds ability to prevent cancer is attributed to its capability to inhibit tumor cell growth, its antioxidant property, and its ability to modulate carcinogen metabolism.

### 4.6. Ferrocenyl–Olefin Derivatives

Oliveira et al. documented the antiproliferative effect of tetrasubstituted olefins–ferrocenyl compounds. In vitro studies were performed on different human cancer cells such as MDA-MB-435 (human melanoma), SF-295 (human glioblastoma), HCT-8 (human colon cancer), and HL-60 (human promyelocytic leukemia). Compound **72a** and **72b** displayed IC_50_ values of 16 µM and 14.2 µM, respectively, and they were found to exhibit moderate antiproliferative activity when compared to other compounds with similar geometry when tested against human breast cancer cell lines MDA-MB-231 (Figure 24) [71]. MDA-MB-435 cell lines was found to be highly sensitive to the compounds with amine side chains at low micromolar activity. The amine side chains contributed to estrogen–receptor interactions.

**Figure 25:**
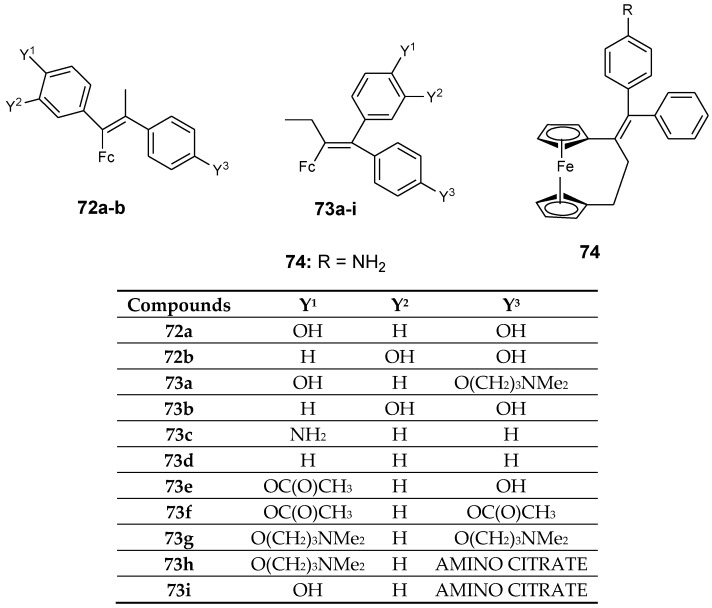
Chemical structures of ferrocenyl–olefins (**72**–**74**).

The introduction of an aromatic substituent did not play an important role in the cytotoxicity activity of the compounds against MDA-MB-231 cells. The presence of the less bulky substituent such as acetyl when compared to a pivaloyl substituent resulted in the enhanced cytotoxic effect of the compounds against the selected cancer cell lines. Compounds **73a**–**i** exhibited good antiproliferative activity with IC_50_ values that are not greater than 2 µM, compound **73a**, **73d**, **73g**, **73h**, **73i** were the most active compounds with inhibition values between 1–2.9 µM for SF-295, 0.82–1.4 µM for HCT-8, 0.83–16 µM for MDA-MB-435, and 0.52–1.9 µM for HL-60, respectively (Figure 25). Compound **73h** exhibited IC_50_ values ranging between 0.52 and 1.9 µM for all human cancer cell lines. Compound **73c**, **73e**, **73f** were slightly active against all human cancer cell lines except MDA-MB-435 with IC_50_ values ranging between 1 and 2.6 µM for SF-295, 1.0–3.0 µM for HCT-8, and 0.90–1.6 µM for HL-60 cancer cell lines. Compound **74** was one of the most antiproliferative compounds against all cancer cell lines except MDA-MB-435 with inhibition values between 0.12–1.0 µM, respectively [71].

**Figure 26:**
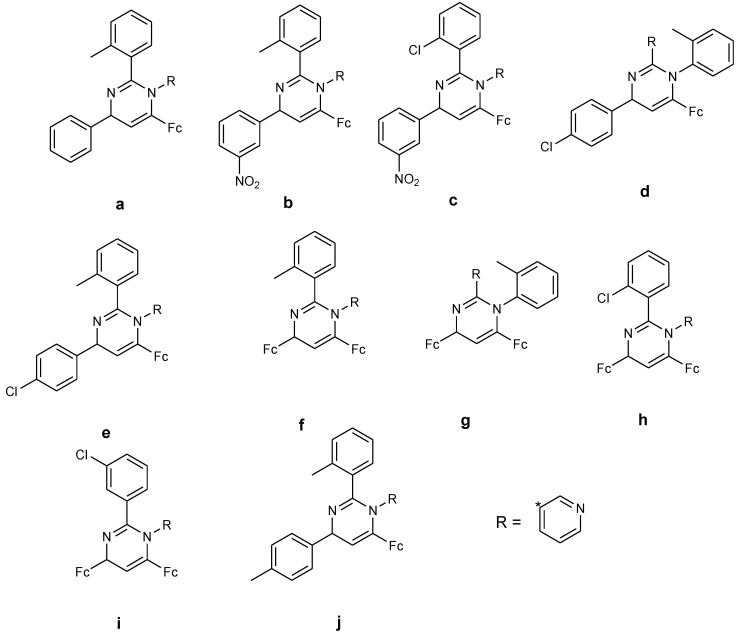
Chemical structures of ferrocene with 1,4-dihydropyrimidine derivatives (**75a**–**j**).

Jadhav et al. synthesized a series of ferrocenyl chalcone and amidines compounds (**75a**–**j**) via catalyzed cyclocondensation and these compounds were tested in vitro for anticancer activity against human breast cancer cell line MDA-MB-435 using the sulforhodamine B assay method (Figure 26). Compounds (**75a**–**g**) exhibited good anticancer activity when compared to doxorubicin (GI_50_= 18.4 µM) against MDA-MB-435 cancer cell lines with GI_50_ values between 16.85–63.2 µM with compound **75b**, **75c**, and **75f** exhibiting low GI_50_ values of 18, 17.4, and 16.85 µM, respectively. Compounds (**75h**–**j**) exhibited poor anticancer activity when compared to doxorubicin with GI_50_ values ranging between 125.5 and 152.9 µM [72]. Compound **75b** and **75c** exhibited high anticancer activity and the presence of a nitro functional group contributed to their anticancer activity.

### 4.7. Ferrocene–Carboxylate Derivatives

**Figure 27:**
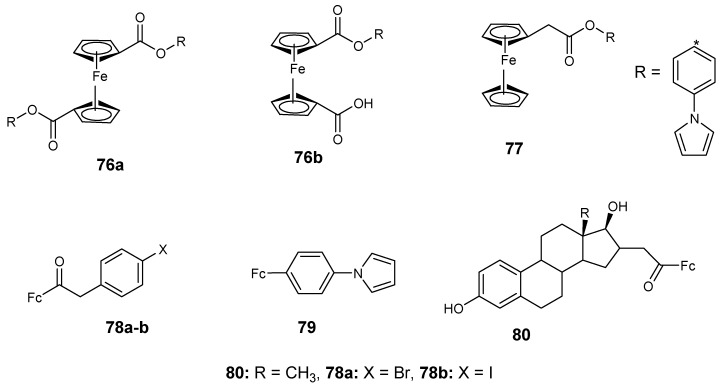
Chemical structures of ferrocene–carboxylate derivatives (**76**–**80**).

Perez et al. reported the synthesis of ferrocene derivatives (**76**–**77**) containing carboxylate and acetylate with anticancer activity and their antiproliferative activity in vitro against MCF-7, MCF-10A, and HT-29 cancer cell lines using an MTT assay was also evaluated (Figure 27). Compound **76a** and **76b** exhibited moderate antiproliferative activity with IC_50_ values of 45.5 and 57 µM. Compound **77** exhibited low antiproliferative activity with an IC_50_ value of 103 µM against MCF-7 breast cancer cell lines. The compounds did not exhibit any significant antiproliferative activity against HT-29 and MCF-10A with inhibition values between 121–298 µM [73].

**Figure 28:**
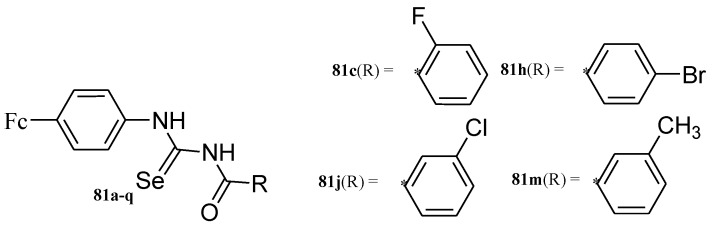
Chemical structures of ferrocene incorporated selenourea derivatives (**81a**–**q**).

Vera et al. reported ferrocene–carboxylate compounds with phenyl and halogen (F, I, Cl, and Br). Compound **78a** exhibited good antiproliferative activity against MCF-7 cancer cells with an IC_50_ value of 9.2 µM and compound **78b** exhibited high antiproliferative activity against MCF-10A cancer cells with an IC_50_ value of 7 µM [74,75] (Figure 27). Compound **79** anticancer activity was significant against both MCF-7 and MCF-10A cancer cells with IC_50_ values 1.4 and 1.6 µM respectively [73,74]. Compound **78a** and **80** showed moderate antiproliferative activity against HT-29 cancer cells with IC_50_ values of 24.4 and 24.0 µM, respectively [73,74] (Figure 28).

The in vitro anti-proliferative studies indicated that the presence of 4-fluorophenyl substituent in the compounds resulted in compounds with no cytotoxic effect against the cancer cell lines. However, the presence of a phenyl group enhanced the proliferative effects of the compound against MCF-7 but nullified the proliferative effect of the compound against the MCF-10A cell line.

### 4.8. Ferrocene Incorporated Selenourea Derivatives

Hussain et al. reported ferrocene incorporated with selenourea derivatives (**81a**–**q**) (Figure 28) and tested them for their anticancer activity against liver cancer (Hepa 1c1c7), breast cancer (MCF-7), and neuroblastoma (MYCN2 and SK-N-SH), in vitro. Only four compounds (**81c**, **81h**, **81j**, and **81m**) exhibited moderate anticancer activity against the aforementioned cells, compound **81c** displayed moderate cytotoxicity activity against MYCN-2 and MCF-7 with inhibition values of 26.5 and 27 µM, compound **81h** exhibited better anticancer activity against MYCN-2 with an inhibition value of 12.2 µM, compound **81j** was slightly cytotoxic with an IC_50_ value of 18.9 µM against MYCN-2 and compound **81m** showed low anticancer activity against MYCN-2 with inhibition value of 38.9 µM [75]. The ortho substitution on the phenyl ring attached with the carbonyl carbon influenced the cytotoxic effect of the compounds significantly. The aromatase inhibition and quinone reductase induction activities of the compounds further revealed their capability to inhibit the cancer initiation and propagation.

### 4.9. Ferrocene–Steroid Conjugates

**Figure 29:**
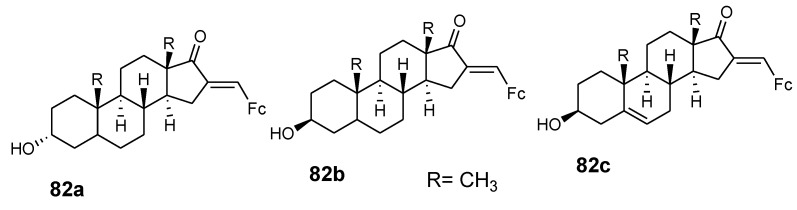
Hybrid compounds **82a**–**c**.

Pita et al. reported the synthesis of ferrocene–steroid conjugates (**82a**–**c**) (Figure 29) and evaluated their anticancer activity against colon cancer HT-29 and breast cancer MCF-7 cell lines. Compounds (**82a**–**c**) exhibited good antiproliferative activity with IC_50_ values less than 30 µM against the MCF-7 breast cancer cell line when compared to tamoxifen with an IC_50_ of 47 µM and 3-estradiol ferrocenecarboxylate with an IC_50_ of 9 µM at low concentration. Compound **82a** showed high antiproliferative activity exceeding that of cisplatin against the HT-29 colon cancer cell line with an inhibition value of 1.2 µM [11]. The presence of ferrocene moiety in the compounds enhanced the anti-proliferative activity of the compound against the HT-29 colon cancer cell line. Estrogen receptor beta play a vital role in the anti-proliferative activity on the HT-29 cell line.

**Figure 30:**
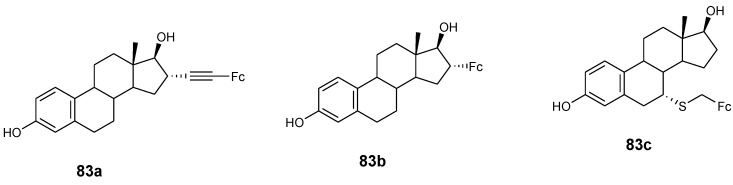
Hybrid compounds **83a**–**c**.

Jaouen and co-workers synthesized ferrocenyl compounds (**83a**–**c**) (Figure 30) and tested them for their anticancer activity in vitro against two human breast cancer cell lines MCF-7 and MDA-MB-231. Compounds **83a**–**c** had no effect on MDA-MB-231 and were estrogenic on the MCF-7 cancer cell line at 0.1–1 µM concentration, compound **83a** and **83c** exhibited cytotoxic activity on MDA-MB-231 at high concentrations with IC_50_ values of 13.4 and 18.8 µM, respectively, while compound **83b** remain inactive [62].

**Figure 31:**
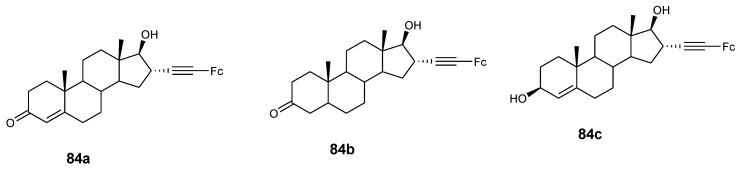
Hybrid compounds **84a**–**c**.

Jaouen et al. also reported the synthesis of steroid vectorized ferrocene derivatives (**84a**–**c**) (Figure 31) and evaluated their antiproliferative effect against prostate cancer cells LNCaP and PC-3 with IC_50_ values ranging between 4.7 and 8.3 µM. At high concentrations (10 µM) compound **84a**–**c** exhibited good antiproliferative activity but poor activity at low concentrations (1 µM) against PC-3 cancer cells. Compound **84a** and **84b** displayed high antiproliferative activity at high concentrations (10 µM), with no activity at low concentrations against LNCaP cancer cells [62].

**Figure 32:**
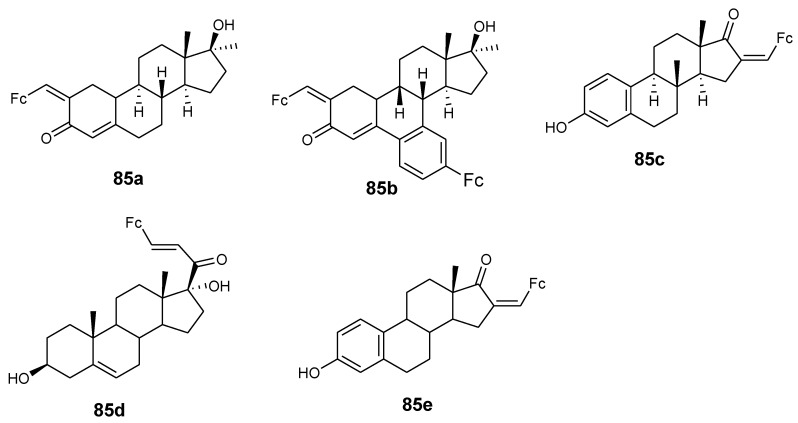
Chemical structures of ferrocene–steroids derivatives (**85a**–**e**).

Manosroi and co-partners synthesized ferrocenic–steroid derivatives (**85a**–**e**) (Figure 32). Firstly, in vitro cytotoxicity evaluation of the parent drug (doxorubicin) against cell line HeLa revealed (GI_50_ value= 0.250 µg/mL) for comparison use. Compound **85a** and **85b** exhibited antiproliferative activity that was comparable with doxorubicin with GI_50_ values of 0.223 and 0.271 µg/mL. Compound **85d** and **85e** displayed moderate antiproliferative activity with GI_50_ values of 0.405 and 0.505 µg/mL while their parent drugs exhibited low antiproliferative activity on HeLa cancer cells [76].

### 4.10. Aminoferrocene-Based Derivatives

**Figure 33:**
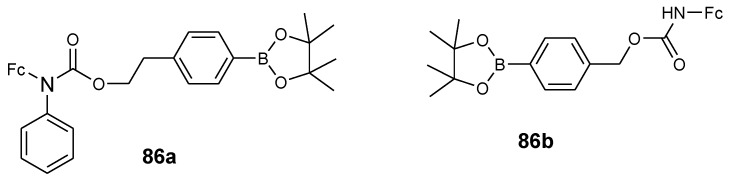
Chemical structure of aminoferrocene-based derivatives (**86a**–**b**).

Schikora et al. reported anticancer activity of aminoferrocene-based compounds (**86a**–**b**) (Figure 33) against LNCaP and DU-145 prostate cancer cell lines. At high concentrations, compound **86b** was found to be non-toxic against DU-145 and LNCaP cancer cells while compound **86a** exhibited moderate cytotoxic activity against both DU-145 and LNCaP cancer cells in vitro with inhibition values that were between 18–27 µM and 11–17 µM, respectively. In vivo studies further revealed that compound **86a** was non-toxic against human prostate adenocarcinoma xenografts in a CBA mice model and exhibited restricted antitumor activity [77]. *N*-benzyl-substituted aminoferrocene-based prodrug 86a exhibited significant anticancer activity against androgen-sensitive human prostate adenocarcinoma cell line LNCaP which is attributed to the increased amount of intracellular reactive oxygen species during the treatment of the cells with the drug and its enhanced cell-membrane permeability when compared to **86b**.

### 4.11. Ferrocene–Lawsone Mannich Derivatives

**Figure 34:**
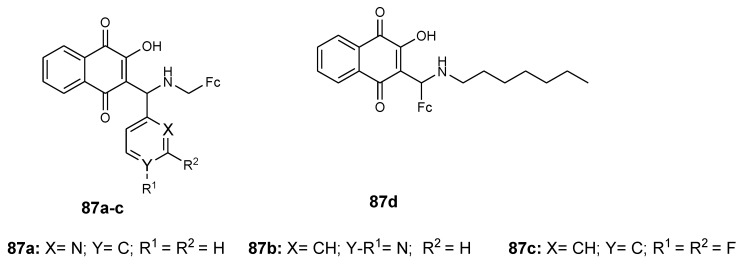
Chemical structures of ferrocene–Lawsone Mannich base derivatives (**87a**–**d**).

Ferrocenyl compounds **(87a**–**d**) (Figure 34) modified by Lawsone Mannich bases were reported by Ahmad et al. Their antiproliferative studies against prostate (BxPC-3), breast (MDA-MB-231), and pancreatic (PC-3) cancer cells indicated that compound **87a** was the most antiproliferative compound in three cancer cells (**87c** < **87d** < **87b** < **87a**), exhibiting high activity when compared to the well-known Lawsone Mannich bases in PC-3 cancer cells. In vitro studies of compound **87a** on three cancer cells (518A2 melanoma, HT-29 colon carcinoma, HCT-116 colon carcinoma, and vinblastine-resistant KB-V1/Vb1 cervix carcinoma) revealed good activity with inhibition values of 2.60, 3.58, 4.29, and 0.19 µM, respectively [78]. The combination of Lawsone with a 2-pyridyl moiety and a ferrocene-1-yl methylamine scaffold in compound **87a** contributed to its anticancer activity. The effect of the ferrocene in compound **87a** was significant.

## 5. Conclusions

Researchers are currently employing different strategies to develop effective drugs for the treatment of malaria and cancer, two chronic conditions. The aim of the development of new antimalarial or anticancer drugs is to overcome drug resistance, toxicity etc. Most of the drugs are developed as hybrid molecules which is a promising approach to develop compounds to overcome drug resistance.

Antimalarial-based hybrid compounds of ferrocene have been extensively studied. Ferroquine, a hybrid molecule prepared from chloroquine and ferrocene exhibit enhanced inhibition effect against beta-haematin formation which makes it more effective than chloroquine. Other quinoline–ferrocene hybrid molecules were prepared and some of the molecules inhibited the efflux mechanism which is common with chloroquine. However, factors such as rigid linkers and the length of the linkers between the parent drugs influenced the antimalarial activity of some of the hybrid molecules significantly. The quinoline moiety was found to influence the transportation of the hybrid molecules to the digestive vacuole of the parasite. However, ferroquine analogues with the presence of a cleavable amide bond did not exhibit significant antimalarial activity. Ferrocene hybrid molecules containing artemisinin were very active with the capability to improve the bioavailability of molecules when compared with artemisinin which is hampered by its poor bioavailability. Other ferrocene hybrid molecules containing either novobiocin, pyrrole, isatin, pyrimidine, and chalcones derivatives also exhibited good antimalarial activity. Factors such as the chain length, nature, and position of the substituent influenced the biological activity of the compounds in vitro.

In vitro and in vivo evaluation of the ferrocene hybrids against several cancer cell lines further revealed the efficacy of ferrrocene hybrid molecules. The length of the non-polar carbon chain, the position of the ferrocenyl group, the nature and position of the substituents in the hybrid molecules, and the dose of the compounds used in the cytotoxicity evaluation were factors that influenced the cytotoxic effect of the compounds.

Several research reports indicate the efficacy of ferrocene-based hybrids. However, there is a pressing need for these molecules obtained so far to be investigated in vivo in order to confirm the results obtained in vitro. However, it is important to develop hybrid compounds that are structurally simpler, synthetically accessible, and less toxic, with enhanced efficacy and progress through all phases of clinical practice, to become available and affordable.

## Figures and Tables

**Table 1:** In vitro activities of compound **1a**–**c** against *P. falciparum* strains (3D7 and W2).

IC_50_ Values (nM)		
**Compound**	**3D7**	**W2**
**1a**	15.4	133.2
**1b**	21.5	30
**1c**	11.7	20.4
Ferroquine	7.8	9.7
Chloroquine	10.6	138.9
